# The correlation between pulmonary fibrosis and methylation of peripheral *Smad3* in cases of pigeon breeder’s lung in a Chinese Uygur population

**DOI:** 10.18632/oncotarget.17763

**Published:** 2017-05-10

**Authors:** Chao Wu, Wei Ding, Qifeng Li, Wenyi Wang, Mingqin Deng, Rong Jin, Baosen Pang, Xiaohong Yang

**Affiliations:** ^1^ Department of Respiratory and Critical Care Medicine, People's Hospital of Xinjiang Uygur Autonomous Region, Urumqi 830001, China; ^2^ Institute of Pediatrics of Xinjiang Uygur Autonomous Region, Urumqi 830001, China; ^3^ Beijing Institute of Respiratory Medicine, Beijing Chao-Yang Hospital, Beijing 100043, China

**Keywords:** *Smad3* gene, pigeon breeder's lung, pulmonary fibrosis, polymerase chain reaction, Uygur

## Abstract

Smad3 is a key protein in the transforming growth factor-beta (TGF-β)/Smad signaling pathway, which is involved in fibrosis in many organs. We investigated the relationship between *Smad3* gene methylation and pulmonary fibrosis in pigeon breeder's lung (PBL). Twenty Uygur PBL patients with pulmonary fibrosis in Kashi between October 2015 and March 2016 were enrolled. Twenty PBL-free pigeon breeders and 20 healthy non-pigeon breeders enrolled during the same period constituted the negative and normal control groups, respectively. Participants’ data and peripheral blood samples were collected, and three *Smad3* CpG loci were examined. Distributions of CpG_2 and CpG_4 methylation rates did not differ across groups, whereas distributions of CpG_3 methylation rates were significantly different among the three groups. The CpG_3 methylation rate was significantly lower in the patient group than in the negative control group. *Smad3* mRNA expression was significantly higher in the patient group than in the negative control group but did not differ between the two control groups. TGF-βlevels were significantly higher in the patient group than in either control group (both P<0.01). *Smad3* gene methylation and *Smad3* mRNA expression were negatively correlated, with a correlation coefficient of -0.84. The number of pigeons bred during the preceding three months was positively correlated with *Smad3* mRNA expression, with a correlation coefficient of 0.77. *Smad3* gene hypomethylation might promote pulmonary fibrosis in Uygur PBL patients via increased *Smad3* mRNA expression. *Smad3* methylation, *Smad3* mRNA expression and TGF-β level were correlated with the number of pigeons bred by patients.

## INTRODUCTION

Pigeon breeder's lung (PBL), which is a form of hypersensitivity pneumonitis (HP) [1], is caused by the inhalation of proteins and other organic particles shed from pigeons (e.g., excrement, feathers, and serum). This induces a strong immune response in pulmonary tissues and causes alveolitis. HP is an interstitial pulmonary disease that commonly results in pulmonary fibrosis [2].

Smad3 is a key protein in the transforming growth factor (TGF)-β/Smad signaling pathway, which plays an important role in the development and progression of fibrosis in many organs [3]. A substantial body of work has demonstrated that the abnormal expression of the *Smad3* gene is an important pathogenic mechanism in diseases such as hepatic, renal, and pulmonary fibrosis [4–6]. Smad3 serves as a critical mediator of fibrotic diseases [7], and abnormal Smad3 expression can influence TGF-β activity, thereby altering the progression of fibrosis [8]. To date, studies concerning the involvement of Smad3 in fibrotic diseases have mainly focused on the TGF-β/Smad pathway, while few studies have reported the gene regulatory mechanisms underlying *Smad3* expression. Tao L proposed that miR-433 may be involved in the pathogenesis of myocardial fibrosis by regulating the expression of Smad3 [9]. However, whether the abnormal expression of Smad3 relates to *Smad* gene methylation has not been determined, and no study has reported whether pulmonary fibrosis in patients with HP involves the Smad3 and the TGF-β/Smad pathway.

China's Xinjiang Uygur population is a typical mixed ethnic group that differs from the Han nationality. This group includes 11 gene types; predominantly Turkic, Arabic, Western European, and Southeast Asian types. The Uygur religion is Islam, and individuals in this group, with the exception of those of mixed race, have long lived in South Xinjiang. We found that there are many Uygur PBL patients in Xinjiang, which may correlate with the frequency of raising pigeons by this group. Approximately 63.6% of known patients with PBL progress to interstitial pneumonia [10]. Previously, we conducted a preliminary screening analysis of whole-genome methylation in 4 cases of Uygur PBL and 4 Uygur pigeon breeders without this disease using the Illumina 450K method (Illumina 450K Infinium Methylation BeadChip; SHBIO, Shanghai, China), and the results showed abnormal methylation of the *Smad3* gene among the Uygur PBL patients.

Given these findings, the objective of this study was to assess a larger sample to further investigate the relationship between *Smad3* gene methylation and pulmonary fibrosis in Uygur PBL patients. To the best of our knowledge, this study is the first investigation to show that pulmonary fibrosis in PBL is associated with *Smad3* and that the abnormal expression of *Smad3* mRNA in PBL is at least partially attributable to gene methylation.

## RESULTS

### General information

This study included 60 subjects divided into three groups, with 20 subjects in each group. The patient group included 11 males and 9 females with an average age of 52.72±12.45 years. The negative control group included 8 males and 12 females with an average age of 53.27±14.22 years. In the normal control group, there were 10 males and 10 females, and the average age was 53.13±11.03 years. There were no statistically significant differences in age or gender composition among the three groups. The extent of pulmonary fibrosis was divided into 6 grades (0-5 points) according to Muller's method. The size of each fibrotic area was evaluated by three radiologists. An evaluation was considered valid when at least two of the radiologists gave similar scores. Otherwise, a re-evaluation was carried out. The general information for the different groups is shown in Table 1 and Table 2.

**Table 1:** General information about research subjects

	Patients with PBL,n=20	Negative controlgroup, n=20	Normal controlgroup, n=20
Age (Years), x¯±s	52.72±12.45	53.27±14.22	53.13±11.03
Gender (Male/Female)	11/9	8/12	10/10
Duration of pigeon breeding			
<3 years	12	0	0
>3 years	8	20	0
Non-pigeon breeding	0	0	20
Number of pigeons kept			
<50	9	0	
>50	11	20	
None	0	0	20
Cigarette smoking			
Yes	0	0	0
No	20	20	20
Area of pulmonary fibrosis lesion (Muller method)			
1 point: lesion area <5%	0	-	-
2 points: 6%-24%	6	-	-
3 points: 25%-49%	8	-	-
4 points: 50%-75%	4	-	-
	2	-	-

**Table 2:** Gender and age ranges of the three groups of research subjects

		Patient group		Negative control		Normal control	
		n	%	n	%	n	%
Gender	Male	11	55.00	8	40.00	10	50.00
	Female	9	45.00	12	60.00	10	50.00
Age (years)	20-40	4	20.00	5	25.00	4	20.00
	40-60	7	35.00	8	40.00	8	40.00
	>60	9	45.00	7	35.00	8	40.00

### *Smad3* methylation

**Figure 1:**
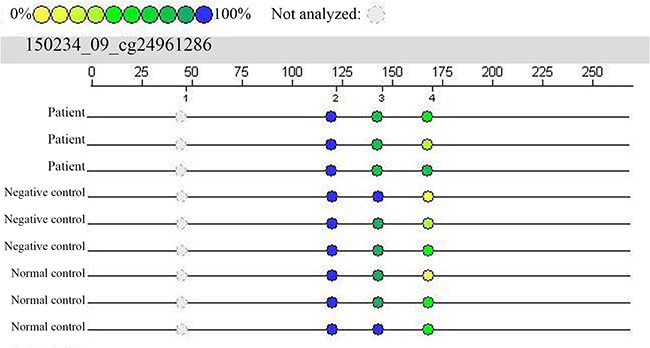
Results from mass spectral analysis of the *Smad3* gene (cg24961286) In this experiment, 4 CpG loci were investigated, and 3 of these loci were captured. In the patient group, CpG_3 is lighter in color, indicating hypomethylation, whereas in the negative and normal control groups, this locus appears darker in color, indicating relatively high methylation.

A total of 4 fragments of the *Smad3* gene (CpG_1, CpG_2, CpG_3, and CpG_4) were investigated, and 3 of them (CpG_2, CpG_3, CpG_4) were captured in our experiment (Figure 1). There were no significant differences in CpG_2 and CpG_4 methylation rates among the three groups (P>0.05), although the distribution of CpG_3 methylation rates differed significantly among the three groups (P<0.05). A pairwise comparison of CpG_3 results among the three groups revealed that the *Smad3* gene methylation rate in the patient group was significantly lower than those in the negative and normal control groups (P<0.017). However, there was no significant difference between the negative control group and the normal control group (P>0.017) (Table 3 and Table 4).

**Table 3:** Medians (P50) and interquartile ranges (P25, P75) of the methylation rate of each CpG locus of the *Smad3* gene in all three groups

CpG	A	B	V	Total
CpG_2	0.94 (0.93,0.96)	0.95 (0.93,0.96)	0.94 (0.92,0.95)	0.94 (0.93,0.96)
CpG_3	0.81 (0.79,0.84)	0.89 (0.85,0.95)	0.85 (0.81,0.89)	0.84 (0.81,0.89)
CpG_4	0.33 (0.06,0.63)	0.39 (0.18,0.63)	0.36 (0.25,0.75)	0.35 (0.04,0.63)

A: Uygur patient group; B: Uygur negative control group; V: Uygur normal control group.

**Table 4:** Statistical comparisons of the methylation rate at each CpG locus for all three groups

CpG site	A/B/V		A/B		A/V		B/V	
	X^2^	P	Z	P	Z	P	Z	P
CpG_2	2.433	0.296	−1.222	0.231	−0.164	0.870	−1.454	0.146
CpG_3	18.288	0.000^*^	−4.261	0.000^*^	−1.873	0.061	−2.360	0.018*
CpG_4	0.148	0.928	−0.380	0.718	−0.272	0.786	−0.054	0.957

A: Uygur patient group; B: Uygur negative control group; V: Uygur normal control group. * denotes P<0.05, and ** denotes P<0.017.

### Smad3 mRNA expression

**Figure 2:**
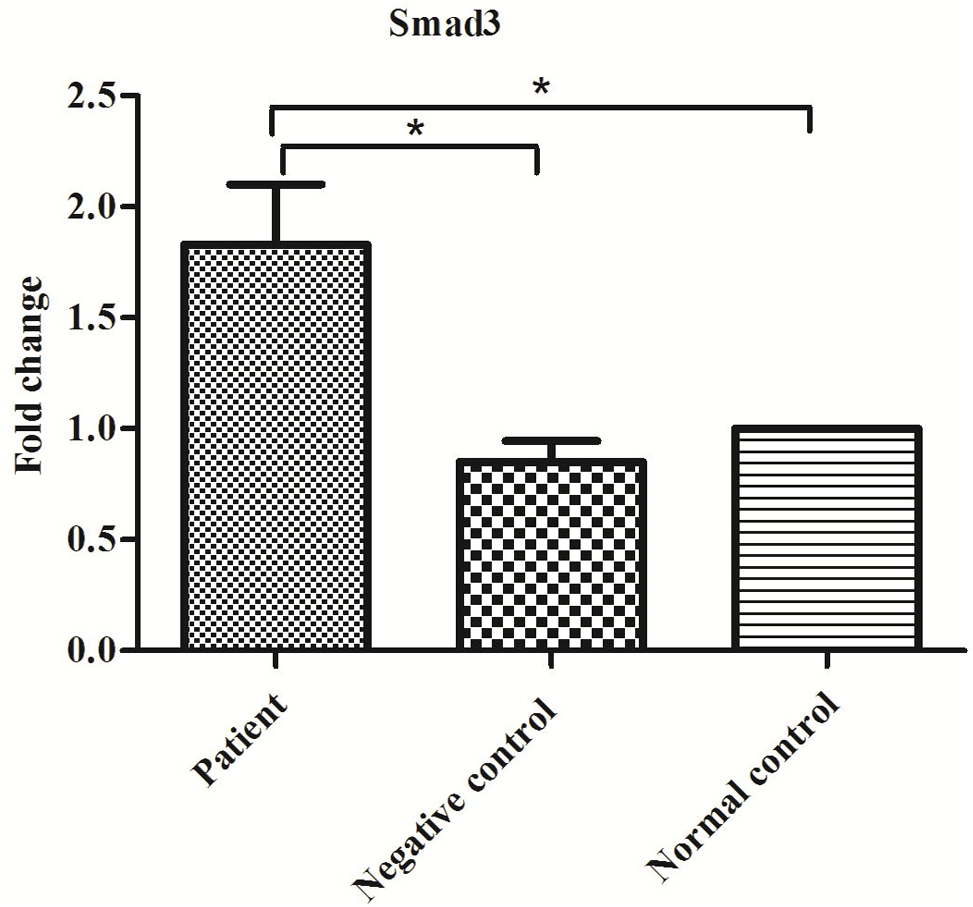
Expression of Smad3 mRNA in the patient, negative control and normal control groups *P<0.05.

The expression of *Smad3* mRNA was detected using fluorescence quantitative PCR, and the results are shown in Figure 2. *Smad3* mRNA expression in the patient group was significantly higher than in the negative control group (P<0.01), although expression showed no significant difference between the negative control and normal groups (P>0.05).

### TGF-β levels

**Figure 3:**
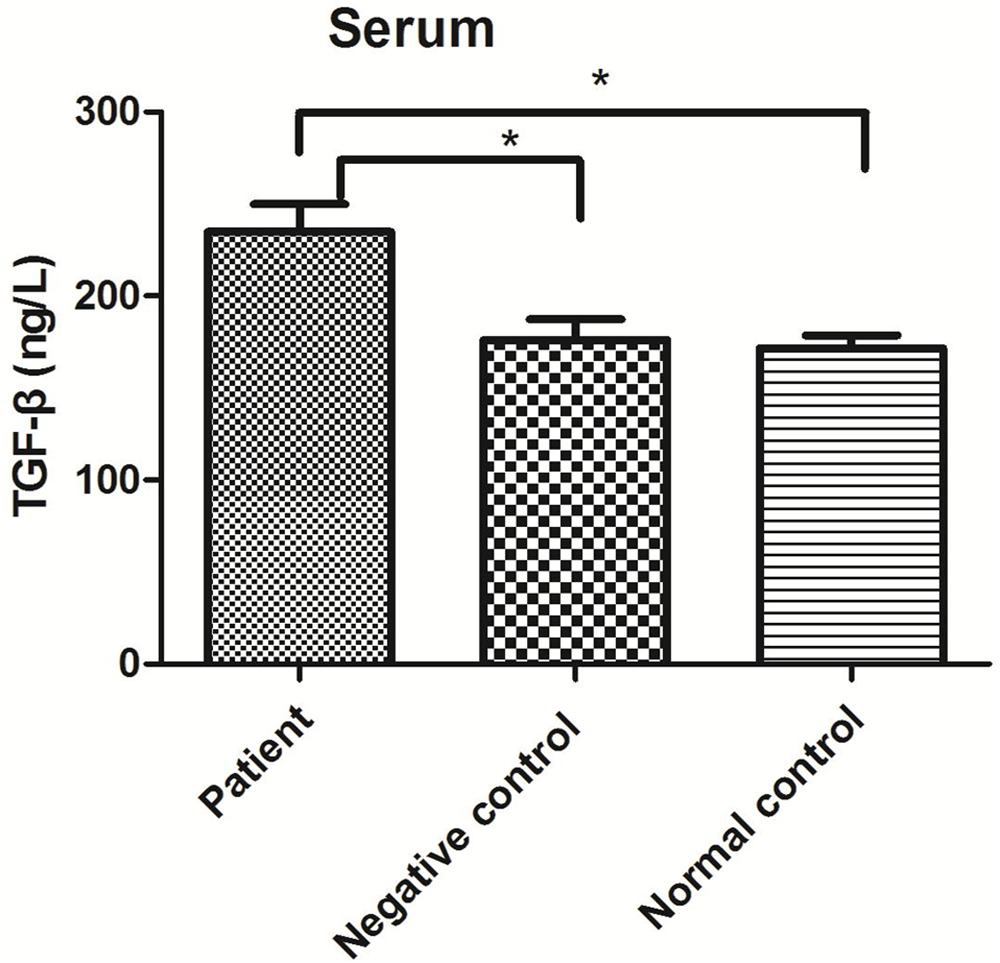
TGF-β levels in serum from the patient, negative control and normal control groups *P<0.05.

**Figure 4:**
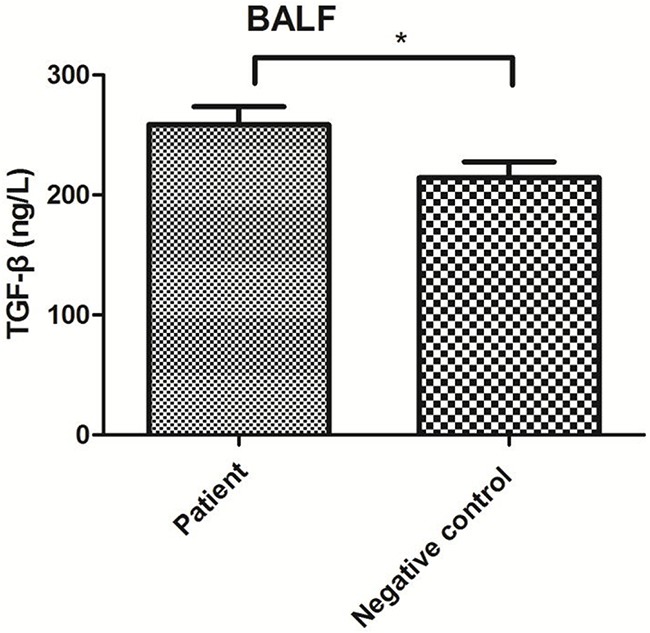
TGF-β levels in BALF from the patient and negative control groups *P<0.05.

TGF-β levels in blood serum and alveolar lavage fluid samples were detected using enzyme-linked immunosorbent assays (ELISAs). TGF-β levels in blood serum were significantly higher in the patient group than in either control group (P<0.01) but did not significantly differ between the control groups (P>0.05; Figure 3). In alveolar lavage fluid samples, TGF-β levels were significantly higher in the patient group than in the negative control group (P<0.01; Figure 4).

### Correlations of *Smad3* gene methylation and *Smad3* mRNA expression with the extent of pulmonary fibrosis

The level of *Smad3* gene methylation, mRNA expression, and the extent of pulmonary fibrosis in patients with PBL were analyzed based on Pearson correlation coefficients. The extent of pulmonary fibrosis was divided into 6 grades (0-5 points) according to Muller's method, where higher values correspond to a larger area of pulmonary fibrosis. In this study, we classified the patients into three groups according to duration of pigeon breeding: < 1 year, 1-3 years, and > 3 years; furthermore, we classified the patients who bred pigeons in the most recent three months according to the number of pigeons bred: <50, 51-100, and >100. *Smad3* gene methylation level was strongly correlated with *Smad3* mRNA expression (correlation coefficient: -0.84), whereas the extent of pulmonary fibrosis was weakly correlated with the *Smad3* gene methylation level and *Smad3* mRNA expression (correlation coefficients: -0.18 and 0.21, respectively). *Smad3* mRNA expression was weakly correlated with pigeon breeding time (correlation coefficient: 0.32) but was more strongly correlated with the number of pigeons bred in the most recent three months (correlation coefficient: 0.77).

## DISCUSSION

PBL belongs to the group of diseases that includes extrinsic allergic alveolitis (EAA), or HP, and is defined as an inflammatory pulmonary disease induced by the repeated inhalation of material shed from pigeons by sensitive individuals [1]. Long-term exposure can lead to pulmonary fibrosis, respiratory failure, pulmonary hypertension, and pulmonary heart disease [12]. Xinjiang is an area with multi-ethnic inhabitants. The Uygur have a tradition of raising pigeons, and coughing and asthma-like symptoms arise repeatedly in certain pigeon breeders; these breeders are often misdiagnosed with asthma, bronchitis, or pneumonia at local hospitals. Only when the condition deteriorates and progresses to pulmonary fibrosis, pulmonary heart disease, or even respiratory failure and seriously affects the patient's life are they transferred to a large hospital and definitively diagnosed. However, at this point, the patients have developed irreversible impairments in their heart and lung function [13]. A survey of pigeon breeder clubs showed that the prevalence of HP in pigeon breeders was approximately 8%-30% [14]. Our survey found that the prevalence of HP in pigeon breeders in Xinjiang was approximately 8%, and most of these breeders developed pulmonary fibrosis because they were not treated in time. After constructing a genomic methylation analysis for patients with PBL and detecting abnormal *Smad3* gene hypomethylation, we hypothesized that pulmonary fibrosis in patients with PBL is correlated with *Smad3* gene methylation.

HP is an interstitial pulmonary disease that commonly develops into pulmonary fibrosis [2]. Lung biopsies have shown that in the acute phase of HP, the following conditions are present: pulmonary interstitial lymphocyte infiltration and fibrosis, edema, non-caseous granulomatosis and bronchiolitis obliterans, and the presence of macrophages with foamy cytoplasm in the interalveolar spaces. In the subacute phase, there is granulomatous inflammation, with lymphocyte, plasma cell, epithelioid cell, and Langerhans cell infiltration, resulting in the thickening of the pulmonary mesenchyme. In the chronic phase, extensive fibrosis becomes prominent and is accompanied by the destruction of the lung parenchyma, collagen deposition and granulation tissue obstruction, which induces bronchial obliterans, and if severe, cystic faveolate changes [15]. At present, a large number of studies have confirmed that the TGF-β/Smad signaling pathway plays an important role in the development and progression of fibrosis in many organs [16]. Smad proteins serve as the main signal transduction factors downstream of TGF-β receptors. Following TGF-β receptor activation, these proteins transmit signals to the nucleus step by step, regulating the proliferation and differentiation of fibroblasts and the activation of target genes responsible for the formation of the extracellular matrix [17].

TGF-β initiates tissue fibrosis progression, and it plays roles in fibroblast proliferation, the differentiation of epithelial cells to fibroblasts, and the synthesis of the extracellular matrix, mainly via the Smad2 and Smad3 signaling pathways [18, 19]. TGF-β binds to the TGF-βI and TGF-βII receptors, activating threonine/serine kinase and promoting Smad2/3 phosphorylation, which then enhances the combination with Smad4. The Smad2/3/4 complex moves to the nucleus and increases the transcription of extracellular matrix genes, thereby enhancing collagen and fibronectin synthesis in the extracellular matrix and promoting the development of pulmonary fibrosis [20, 21]. This study showed that in the patient group, the *Smad3* gene was abnormally hypomethylated compared with its status in the negative control group, *Smad3* mRNA was highly expressed, and TGF-β levels in blood serum and alveolar lavage fluid were elevated. These results are consistent with findings on pulmonary fibrosis, liver fibrosis, and other fibrotic diseases reported in the literature [22, 23].

PBL is a type of HP that eventually progresses to pulmonary fibrosis. One study suggested that TGF-β pathway-induced fibrosis is mediated by Smad3 and that TGF-β/Smad3 is the main pathway regulating the differentiation of myofibroblasts [24]. We found that Xinjiang Uygur pigeon breeders mainly live in south Xinjiang, in areas such as Kashi, Hotan, Aksu and other regions, where keeping pigeons is a source of income for local farmers. Due to a lack of knowledge of this disease, most patients with PBL receive delayed diagnoses in larger hospitals at a time when PBL has progressed to pulmonary fibrosis. Additionally, PBL treatment requires the patient to avoid allergens and receive oral glucocorticoid therapy. However, pigeon breeding is a population-level phenomenon; even if the patients themselves do not breed pigeons, there are no effective measures to keep them away from allergens. As a result, most patients have poor outcomes even after drug therapy as they are continually exposed to allergens. This continual exposure is the main reason for the large number and greater severity of pulmonary fibrosis cases among Uygur PBL patients. Our study found that *Smad3* was involved in the development of pulmonary fibrosis in patients with PBL and that *Smad3* gene methylation was the cause of abnormal *Smad3* mRNA expression. Previous studies, both in China and in other countries, have mainly focused on the relationship between *Smad3* and fibrosis, and no prior investigation has reported the abnormal expression of *Smad3* with respect to methylation of the *Smad3* gene. In this study, we found that abnormal *Smad3* mRNA expression was due to its methylation status, which has significance for the pathogenesis and treatment of PBL. As *Smad3* mRNA increased, TGF-β levels in serum and alveolar lavage fluid also increased. Smad3 serves as a downstream molecule in the TGF-β/Smad signaling pathway. Therefore, *Smad3* gene hypomethylation may be caused by long-term exposure to allergen sources; an increase in levels of TGF-β, an upstream factor of Smad3; or interactions between these two factors. Nevertheless, the causality between the increase in TGF-β and *Smad3* hypomethylation remains to be explored.

Our results show that *Smad3* mRNA expression is negatively correlated with its degree of methylation and is positively correlated with the number of pigeons bred in the most recent three months but is only poorly correlated with the extent of interstitial pulmonary fibrosis and the time spent breeding pigeons. Gene methylation is an important mechanism of gene regulation, and hypermethylation can lead to reduced gene expression. In contrast, hypomethylation can lead to increased gene expression [25]. *Smad3* mRNA expression levels were associated with the level of exposure to allergens and disease progression, in which the number of pigeons bred represents exposure (it determines the severity of inflammatory reactions), but the extent of pulmonary fibrosis may not represent the degree of inflammation in patients. In this study, 20 cases of PBL were selected first after screening using a questionnaire survey of pigeon breeders conducted by our research group in the Kashi region, and diagnoses were confirmed via additional methods, such as lung computed tomography (CT) and pulmonary function tests. However, because of the sample size was small, we could not explain the difference in methylation observed between the three groups even though we found hypomethylation in the patient group relative to methylation in the negative control group; this finding needs to be validated in a larger sample size.

This study is the first investigation to demonstrate that the *Smad3* gene might participate in the development of pulmonary fibrosis in Uygur PBL via increased mRNA expression induced by hypomethylation. However, further studies are needed to explore the correlation between pulmonary fibrosis in Uygur PBL and the TGF-β pathway.

## MATERIALS AND METHODS

### Research subjects

From October 2015 to March 2016, we performed a survey in Kashi, Xinjiang. Twenty Uygur PBL patients with pulmonary fibrosis, who were diagnosed at the First People's Hospital of Kashi or the People's Hospital of Xinjiang Uygur Autonomous Region, were included as the patient group. The patients were 20-80 years old and of either gender. Diagnoses of PBL were based on the diagnostic criteria proposed by Schuyler et al. [10]. Scores for the extent of pulmonary fibrotic lesions in patients with PBL were >1 point based on the “Muller method”. The exclusion criteria included the following: patients with interstitial lung disease, chronic bronchitis, acute respiratory distress syndrome, autoimmune diseases, and malignant tumors; patients with other allergic diseases and other diffuse lung diseases; and those breeding pigeons for less than 1 year or keeping other birds.

Additionally, 20 Uygur pigeon breeders with 3 years of ownership history and without PBL over the corresponding period were selected as the negative control group. Twenty cases of healthy Uygur non-pigeon breeders were selected as the normal control group.

This study was approved by the Ethics Committees of the First People's Hospital of Kashi and the People's Hospital of Xinjiang Uygur Autonomous Region (no. 2012048). Informed consent was obtained from all participants.

### Methods

#### Sample collection

General data were collected from each research subject by having them complete a survey form that included questions about, gender, age, smoking history, and history of pigeon contact. In addition, clinical data, laboratory findings, pulmonary function data, and radiographic data were collected. Concurrently, 2 tubes of fasting peripheral venous blood were drawn from each research subject and mixed with the anticoagulant sodium citrate. After centrifugation, white blood cells were separated and stored at -80°C. mRNA was measured from 1 sample within 1 month after treatment with RNAlater.

#### DNA preparation

DNA was extracted from the blood samples using the Wizard Genomic DNA purification kit (Promega; Madison, WI, USA). Quantitative analyses were performed using a spectrophotometer, while qualitative assessments were performed via agarose gel electrophoresis; the genomic DNA electrophoresis band is usually not less than 20 kb. Qualified DNA were adjusted to a concentration of 50 ng/μl, transferred to 384-well plates, and stored at -20°C for later use.

#### RNA preparation

Total RNA from the blood samples was extracted using the Qiagen RNeasy Mini Kit (Qiagen; Düsseldorf, Germany), and reverse transcribed to cDNA using the Transcriptor cDNA Synthesis Kit (Roche; Basel, Switzerland).

#### Primer design

Primers for *Smad3* gene methylation sites were designed and synthesized by Boao Biological Co., Ltd., Beijing, China. The *Smad3* gene fragment length was 250 bp. The *Smad3* gene fragment sequence was as follows: AAGCAATGCTTTAAGCTTGGTCTAGCTCCCAACAACGAGGCCAAGGCATGGATTCCCTGGTGGAAGTACAAGCTACCAGTGTCTACCAGAAAACTAAGTTTTAAGTGTGCCGAGCTCCTTATTCCTCATATAACGCTCACACACCAACTCTCATTCAGCGGCAAATGCTTTGATTCCATTCTTATAAACTGGCAAGGAAGATACCTGCAGCTTTACTCAGGTACTCAAGCAGGTGAGGCATGAGAGGCTG. The primers and their sequences are shown in Table 5.

**Table 5:** Primers used for PCR amplification and their sequences

Primer	Sequence of primer
Smad3 upstream primer	AAGTAATGTTTTAAGTTTGGTTTAGTTTTT
Smad3 downstream primer	CAACCTCTCATACCTCACCTACTTA
Smad3 modified upstream primer	aggaagagagAAGTAATGTTTTAAGTTTGGTTTAGTTTTT
*Smad3* modified downstream primer	cagtaatacgactcactatagggagaaggctCAACCTCTCATACCTCACCTACTTA

Primers were designed using DNAMAN software to investigate *Smad3* mRNA expression levels. The primer sequences were 5′-ATGGGCTCCTCATTTCAC-3′ (upstream) and 5′-AGCCTCTTTGGATGTCTTCT-3′ (downstream). The length of the amplified product was 133 bp.

#### PCR amplification

PCR amplification was performed using a PCR amplification kit (San Diego, CA, USA) manufactured by SEQUENOM, Inc. with the following conditions. The reaction was started at 94°C for 12 min. Cycle 1 involved 10 cycles of 93°C for 50 sec; 62°C for 48 sec, with this temperature lowered by -0.5°C/cycle; and 72°C for 1 min. Cycle 2 was performed 35 times at 94°C for 45 sec, followed by 56°C for 48 sec and 72°C for 1 min. The final step was conducted at 72°C for 3 min, and the reaction product was then maintained at 4°C. After the above procedures were completed, the amplified products were subjected 2% agarose gel electrophoresis and then qualitatively measured.

#### Alkaline phosphatase (SAP) treatment

This step was performed using the MassCLEAVE Kit (SEQUENOM, USA).

#### In vitro transcription and ribonuclease (RNase) digestion

The MassCLEAVE Kit (SEQUENOM, USA) was used for this step, strictly following the instructions provided with the kit.

#### Chip sample loading and mass spectrometry

The purified product was applied to a 384 SpectroCHIP chip using a MassARRAY Nanodispenser RS1000 arrayer (SEQUENOM, USA), and the chip was analyzed using the MassARRAY Compact System (SEQUENOM). Matrix-assisted laser desorption/ionization-time of flight-mass spectrometry (MALDI-TOF-MS) was used to analyze the SpectroCHIP chip. The data were analyzed and the results outputted using EpiTYPER software (SEQUENOM, USA).

#### Real-time fluorescence quantitative PCR analysis

A quantitative analysis of *Smad3* mRNA expression was conducted using a QuantiFast SYBR Green PCR Kit (Qiagen).

#### ELISA analysis

TGF-β levels in blood serum and alveolar lavage fluid samples were determined using ELISAs, which were performed strictly in accordance with instructions provided with the ELISA kit (Diaclone, France).

### Statistical analysis

The experimental data were analyzed with SPSS 17.0 statistical software. The Kruskal–Wallis test was employed to compare the methylation rates of the three groups. Differences were regarded as statistically significant if P<0.05 (α=0.05). When using a Mann–Whitney test to make pairwise comparisons among three groups, the probability of a type I error increases due to repeated hypothesis testing. Therefore, the test level was corrected to α'=0.017, with differences reaching statistical significance when P<0.017. *Smad3* mRNA expression levels and serum TGF-β levels among the three groups were compared using single-factor analysis of variance, with P<0.05 regarded as statistically significant. Pairwise comparisons among the three groups were made using the SNK-q test. To evaluate TGF-β levels in alveolar lavage fluid samples, a *t* test was performed, and P<0.05 was considered statistically significant. Pearson correlation coefficients were used to assess correlations, and GraphPad Prism 6 was used to plot results.

## Abbreviations

TGF: transforming growth facto
PBL: pigeon breeder's lung
HP: hypersensitivity pneumonitis
EAA: extrinsic allergic alveolitis
CT: computed tomography
SAP: alkaline phosphatase
